# bHLH transcription factor bHLH115 regulates iron homeostasis in *Arabidopsis thaliana*

**DOI:** 10.1093/jxb/erx043

**Published:** 2017-03-28

**Authors:** Gang Liang, Huimin Zhang, Xiaoli Li, Qin Ai, Diqiu Yu

**Affiliations:** 1 Key Laboratory of Tropical Plant Resources and Sustainable Use, Xishuangbanna Tropical Botanical Garden, Chinese Academy of Sciences Kunming, Yunnan 650223, China; 2 University of Chinese Academy of Sciences, Beijing 100049, China; 3 School of Life Sciences, University of Science and Technology of China, Hefei, Anhui 230027, China

**Keywords:** Arabidopsis, Fe deficiency, bHLH115, BTS, iron, PYE.

## Abstract

Iron (Fe) deficiency is a limiting factor for the normal growth and development of plants, and many species have evolved sophisticated systems for adaptation to Fe-deficient environments. It is still unclear how plants sense Fe status and coordinate the expression of genes responsive to Fe deficiency. In this study, we show that the bHLH transcription factor bHLH115 is a positive regulator of the Fe-deficiency response. Loss-of-function of *bHLH115* causes strong Fe-deficiency symptoms and alleviates expression of genes responsive to Fe deficiency, whereas its overexpression causes the opposite effect. Chromatin immunoprecipitation assays confirmed that bHLH115 binds to the promoters of the Fe-deficiency-responsive genes *bHLH38*/*39*/*100*/*101* and *POPEYE* (*PYE*), which suggests redundant molecular functions with bHLH34, bHLH104, and bHLH105. This is further supported by the fact that the *bhlh115-1* mutant was complemented by overexpression of any of *bHLH34*, *bHLH104*, *bHLH105*, and *bHLH115*. Further investigations determined that bHLH115 could interact with itself and with bHLH34, bHLH104, and bHLH105. Their differential tissue-specific expression patterns and the severe Fe deficiency symptoms of multiple mutants supported their non-redundant biological functions. Genetic analysis revealed that bHLH115 is negatively regulated by BRUTUS (BTS), an E3 ligase that can interact with bHLH115. Thus, bHLH115 plays key roles in the maintenance of Fe homeostasis in *Arabidopsis thaliana*.

## Highlight

bHLH115 functions downstream of the E3 ligase BRUTUS and positively controls the expression of the iron-deficiency responsive bHLH transcription factors *POPEYE* and *bHLH38*/*39*/*100*/*101* for iron homeostasis in Arabidopsis.

## Introduction

Iron (Fe) is an important micronutrient for plant growth and development. Its role as a cofactor in the reduction–oxidation reaction makes it indispensable for many essential protein and enzymatic processes, including photosynthesis and respiration (Marschner, 1995; de Benoist *et al.*, 2008). Although Fe is abundant, it mainly exists in the form of insoluble ferric oxides not directly available to plants; this is especially the case in aerobic or alkaline soils (Guerinot and Yi, 1994). Iron deficiency can limit plant growth, and subsequently reduces crop yield and quality (Guerinot and Yi, 1994; Marschner, 1995; Jeong and Guerinot, 2009; Kobayashi and Nishizawa, 2012). Iron overload can also be toxic; this is due to the production of hydroxyl radicals via the Fenton reaction. Therefore, the maintenance of Fe homeostasis is crucial for plant growth and development.

There are two major ways in which plants acquire Fe from the soil. To overcome iron-limiting conditions, non-graminaceous (strategy I) plants employ a reduction strategy to acquire Fe from soil (Hell and Stephan, 2003; Walker and Connolly, 2008; Hindt and Guerinot, 2012), whereas graminaceous plants utilize strategy II, in which phytosiderophores are released into the rhizosphere to chelate Fe (Walker and Connolly, 2008; Hindt and Guerinot, 2012). Strategy I involves three processes: rhizosphere acidification, reduction, and uptake. Upon iron deficiency, H^+^-ATPases pump protons out of the cells to reduce the rhizosphere pH, thus improving solubility of ferric Fe (Santi and Schmidt, 2009). Following acidification, the plasma membrane-bound FERRIC REDUCTION OXIDASE 2 (FRO2) reduces Fe on the root surface from ferric to ferrous Fe (Robinson *et al.*, 1999). Finally, the high-affinity ferrous Fe transporter IRON-REGULATED TRANSPORTER 1 (IRT1) takes up ferrous Fe into the root epidermis (Henriques *et al.*, 2002; Varotto *et al.*, 2002; Vert *et al.*, 2002). Fe absorbed by the roots is chelated with citrate or nicotianamine, and subsequently these complexes are translocated to other tissues and organs (Rogers and Guerinot, 2002; Haydon and Cobbett, 2007; Curie *et al.*, 2009).

The regulation of transcription factors is essential for accurate control of Fe homeostasis. Several transcription factors have been characterized as regulating Fe homeostasis. *FIT* (*FER-like Iron-deficiency-induced Transcription Factor*) encodes a bHLH protein, and is a homologous gene of *FER* in tomato (*Solanum lycopersicum*) (Ling *et al.*, 2002; Colangelo and Guerinot, 2004; Yuan *et al.*, 2005). *FIT* is induced in the epidermis of the root upon iron deficiency (Colangelo and Guerinot, 2004). The *fit* mutant is chlorotic, and its seedling can not survive without an extra Fe supply. Expression of the Fe uptake-associated genes *IRT1* and *FRO2* is greatly reduced in the *fit* mutant (Colangelo and Guerinot, 2004). Overexpressing *FIT* alone does not increase *FRO2* and *IRT1* expression unless *FIT* is co-expressed with one of the bHLH subgroup Ib members (*bHLH38*/*39*/*100*/*101*) (Colangelo and Guerinot, 2004; Yuan *et al.*, 2008; Wang *et al.*, 2013). FIT and bHLH38/39/100/101 function as positive regulators of *IRT1* and *FRO2*. Another bHLH family member, POPEYE (PYE), participates in Fe homeostasis by negatively regulating the expression of *NICOTIANAMINE SYNTHASE 4* (*NAS4*), *FRO3*, and *ZINC-INDUCED FACILITATOR1* (*ZIF1*) (Long *et al.*, 2010). Recent studies suggest that bHLH34, bHLH104, and bHLH105 play key roles in maintaining Fe homeostasis by positively regulating *bHLH38*/*39*/*100*/*101* and *PYE* (Zhang *et al.*, 2015; Li *et al.*, 2016).

Plants can sense internal and external Fe concentrations and they modulate gene expression to maintain intercellular Fe homeostasis. In mammals, FBXL5 (F-BOX AND LEUCINE-RICH REPEAT PROTEIN 5) was identified as a Fe sensor. FBXL5 contains a hemerythrin domain (HHE) for Fe binding, and an F-box domain for ubiquitination and degradation of IRP2 (IRON REGULATORY PROTEIN2), which participates in the regulation of Fe homeostasis (Salahudeen *et al.*, 2009; Vashisht *et al.*, 2009). Similar to FBXL5, BTS (BRUTUS) in Arabidopsis possesses HHE domains; these bind to the Fe ion and RING domains present in many E3 ligases (Selote *et al.*, 2015). Moreover, BTS has a ubiquitination function and binds Fe –hence it is a potential Fe sensor. BTS interacts with PYE-like proteins (bHLH105/ILR3, bHLH104, and bHLH115) and targets bHLH105 and bHLH115 for 26S proteasome-mediated degradation (Selote *et al.*, 2015). Consistent with these observations, BTS negatively regulates Fe homeostasis (Selote *et al.*, 2015; Zhang *et al.*, 2015). Despite the degradation of bHLH115 by BTS, it remains unclear how bHLH115 participates in the regulation of Fe homeostasis.

In this study, we investigated the function of bHLH115 by using a reverse genetic method. *bhlh115* mutants displayed enhanced sensitivity to Fe deficiency, whereas overexpression of *bHLH115* promoted Fe accumulation. Consistent with this, bHLH115 positively regulated expression of Fe-deficiency-responsive genes. Further analyses indicated similar molecular functions and different biological functions among bHLH subgroup IVc members. Genetic analysis also confirmed negative regulation of bHLH115 by BTS.

## Materials and methods

### Plant materials and growth conditions

The *Arabidopsis thaliana* ecotype Col-0 was used in this study. T-DNA insertion lines for *bhlhl115-1* (WiscDsLox384C11) and*bhlh115-2* (WiscDsLox384D9) were sourced from the Arabidopsis Biological Resource Center (ABRC; https://abrc.osu.edu/) and were confirmed using PCR with a T-DNA primer (p745) and gene-specific primers (see Supplementary Table S1 at *JXB* online). The *bhlh34*, *bhlh104*, *bhlh105*, and *bts-1* mutants have been described previously (Long *et al.*, 2010; Li *et al.*, 2016). The homozygous mutants were crossed with each other to generate double- and triple-mutants. All knockout lines were confirmed by PCR analysis.

For phenotypic analyses of the Fe deprivation response, seeds were surface-sterilized and vernalized for 2–4 d at 4 °C before being plated on Murashige and Skoog (MS) medium. Fe-sufficient (+Fe) medium was half-MS medium with 1%sucrose, 0.8% agar, and 0.1 mM FeEDTA. Fe deficient (–Fe) medium was the same but without iron. For –Fe +Frz media, Fe-deficient (–Fe) medium was supplied with 0.05mM ferrozine. Plates were placed in a culture room at 22 °C under a 16-h light/8-h dark photoperiod.

### Plasmid construction and plant transformation

Standard molecular biology techniques were used for the cloning procedures. Genomic DNA from Arabidopsis ecotype Col-0 was used as the template for amplification of the upstream regulatory promoter sequence for *ProbHLH115:GUS*. For the generation of *bHLH115-OX* transgenic plants, one HA tag-fused bHLH115 open reading frame was cloned into the binary vector pOCA30 in the sense orientation behind the CaMV 35S promoter. The *bHLH105-OX* construct was constructed by a similar method. *bHLH34-OX* and *bHLH104-OX* have been described previously (Li *et al.*, 2016). *Agrobacterium tumefaciens* strain EHA105 was used for transformation into Col-0 plants through the floral dipping method (Clough and Bent, 1998). Transgenic plants were selected on half-strength MS agar plates with 50 µg ml^–1^ kanamycin.

### Gene expression analysis

One microgram of total RNA extracted using the Trizol reagent (Invitrogen) was used for oligo(dT)_18_ primed cDNA synthesis according to the reverse-transcription protocol (Fermentas). The resulting cDNA was subjected to relative quantitative PCR using a SYBR Premix Ex Taq^TM^ kit (TaKaRa) on a Roche LightCycler 480 real-time PCR machine, according to the manufacturer’s instructions. *ACTIN2* (*ACT2*) was amplified as an internal control, and gene copy number was normalized to that of *ACT2*. For the quantification of each gene, at least three biological replicates were used. Each biological replicate contained three technical replicates. Analysis for statistical significance was performed using Student’s *t*-test. The qRT-PCR primers have been described previously (Li *et al.*, 2016).

### Histochemical GUS staining

GUS staining of *ProbHLH115:GUS* was performed using the following procedure. Briefly, fresh seedlings were fixed in ice-cold 90% (v/v) acetone for 20 min and then vacuum-filtrated for 10 min with staining solution containing 0.5 mg ml^–1^ 5-bromo-4-chloro-3-indolyl-b-gl-ucuronic acid (Sigma) in 0.1M sodium phosphate buffer (pH 7.3). Then the samples were incubated in the dark at 37 °C until a blue-indigo color appeared. After the reaction, seedlings were rinsed in 0.1M sodium phosphate buffer (pH 7.3). The samples were then rinsed twice in 70% ethanol to remove chlorophylls.

### Measurement of Fe content

Seeds were germinated and grown on iron-sufficient media for 10 d. Then fresh shoot samples were harvested separately and dried for 3d in an oven at 65 °C. Analysis of Fe content was performed using inductively coupled plasma spectroscopy. Means and standard deviations were calculated from three biological replications.

### Ferric chelate reductase assays

Ferric chelate reductase assays were performed as previously described (Yi and Guerinot, 1996) with some modifications. Briefly, seeds were germinated, and grown on iron-sufficient media for 4 d, and then transferred to iron-sufficient or -deficient plates for 3 d. Roots of 10 seedlings were then weighed and placed in assay solution containing 0.1 mM Fe(III)-EDTA and 0.3 mM ferrozine. The roots were incubated in the dark for 20 min. An identical assay solution containing no plants was used as a blank. The purple-colored Fe(II)-ferrozine complex was measured at 562nm using a molar extinction coefficient of 28.6 mM^–1^ cm^–1^.

### Chlorophyll measurement

Chlorophyll content was measured in 2-week-old plants grown on Fe-sufficient and Fe-deficient media. All leaves are collected and ground to powder in liquid nitrogen. The powder was resuspended in 80% acetone on ice and centrifuged for 5 min at 10000 *g* at 4 °C. Chlorophyll concentrations were calculated from spectroscopy absorbance measurements at 663.2, 646.8, and 470 nm (Lichtenthaler, 1987).

### Chromatin immunoprecipitation (ChIP)assays

ChIP assays were performed essentially according to previously described protocols (Saleh *et al.*, 2008). Wild-type and *bHLH115-OX-14* transgenic plants grown on –Fe media for 2 weeks were used for ChIP assays. To quantify bHLH115-DNA binding, qPCR was performed according to the procedure described previously with the *TUBIN2* as the endogenous control (Mukhopadhyay *et al.*, 2008). All primers are listed in Supplementary Table S1. For the quantification of each DNA fragment, three biological replicates were used. Each biological replicate contained three technical replicates.

### Transient expression assays

*Agrobacterium tumefaciens* strain EHA105 was used to infect tobacco leaves. Agrobacterial cells containing plasmids were infiltrated into leaves of *Nicotiana benthamiana* by an infiltration buffer (0.2 mM acetosyringone, 10 mM MgCl_2_, and 10 mM MES, PH 5.6). For the bimolecular fluorescent complementation (BiFC) assays, equal volumes of an *Agrobacterium* culture were mixed before infiltration into *N. benthamiana* leaves. After infiltration, yellow fluorescence protein (YFP) and DAPI fluorescence were observed under a confocal laser scanning microscope (Olympus, Tokyo, Japan). For the transcription activation assay, the final OD600 values were 0.1 (an internal control; *Pro*_*35S*_*:Myc-GUS*), 0.5 (reporter), and 0.5 (effector). After infiltration, plants were placed in the dark at 24 °C for 48 h before RNA extraction. The transcript abundance of *GFP* was normalized to *GUS.*

### Yeast assays

For the yeast two-hybrid (Y2H) assay, the N-terminal truncated *bHLH115N* containing the bHLH domain was cloned into pGBKT7, and the full-length ORF of *bHLH115*was cloned into pGADT7. GAD-bHLH34, GAD-bHLH104, and GAD-bHLH105 have been described previously (Li *et al.*, 2016). The combination of pGBKT7-53 and pGADT7-T was used as a positive control and the combination of pGBKT7-Lam and pGADT7-T as a negative control. Growth was determined as described in the Yeast Two-Hybrid System User Manual (Clontech). Primers used for the vector construction are listed in Supplementary Table S1. Experiments were repeated three times.

### Accession numbers

Sequence data generated in this study have been lodged in the Arabidopsis Genome Initiative and GenBank/EMBL databases under the following accession numbers: bHLH34 (At3g23210), bHLH104 (At4g14410), bHLH105 (At5g54680), bHLH115 (At1g51070), bHLH38 (At3g56970), bHLH39 (At3g56980), bHLH100 (At2g41240), bHLH101 (At5g04150), FIT (At2g28160), MYB10 (At3g12820), MYB72 (At1g56160), FRO2 (At1g01580), IRT1 (At4g19690), PYE (At3g47640), BTS (At3g18290), and ACT2 (At3g18780).

## Results

### *Loss-of-function of* bHLH115 *alters response to Fe deficiency*

**Fig. 1.**
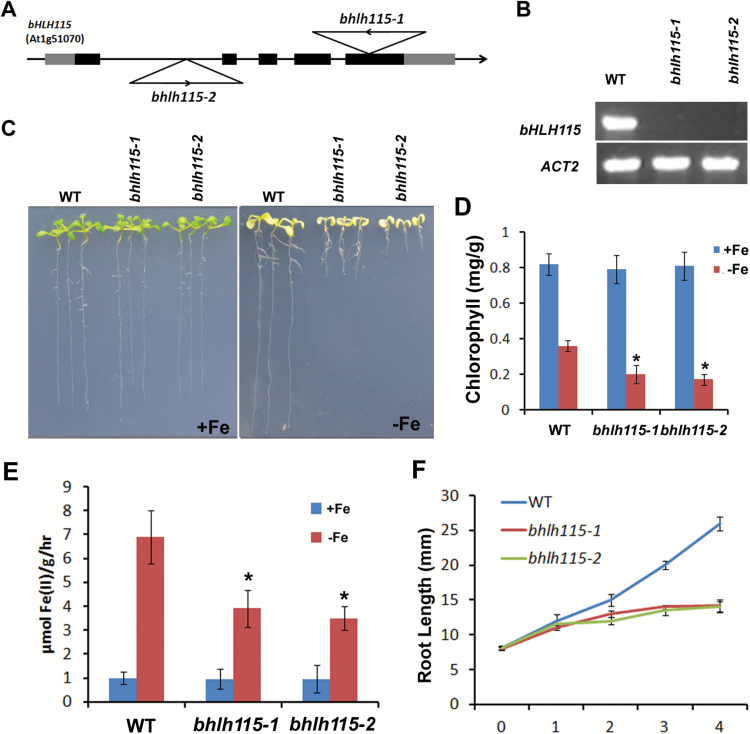
Characterization of *bhlh115* mutants. (A) Location of T-DNA in the *bHLH115* gene. Thick bars and lines indicate the exons and introns, respectively. Grey bars indicate the 5′ and 3′ untranslated regions. Triangles indicate the location of the T-DNA. (B) Confirmation of the *bhlh115-1 and bhlh115-2* mutants. The full-length cDNA of *bHLH115* was amplified by RT-PCR. (C)Images of 10-d-old seedlings that were germinated directly on +Fe or –Fe media. (D) Chlorophyll content of leaves on +Fe or –Fe media. Significant differences from the wild-type were determined by Student’s *t*-test, *P<0.05). (E) Iron reductase activity. Seedlings were germinated and grown on +Fe medium for 10 d and then transferred to –Fe media for 3 d. Significant differences from the wild-type were determined by Student’s *t*-test, *P<0.05). (F) Time-course quantification of root length of plants from 0 to 4 d after transfer to –Fe medium.

Three members of the bHLH subgroup IVc (Heim *et al.*, 2003), bHLH34, bHLH104, and bHLH105, regulate Fe homeostasis (Zhang *et al.*, 2015; Li *et al.*, 2016); however, the functions of the other member, bHLH115, are not clear. We therefore screened two T-DNA insertion lines of the *bHLH115* gene sourced from the Arabidopsis Biological Resource Center (Fig. 1A). Two homologous mutants, *bhlh115-1* and *bhlh115-2*, were identified as null mutants, exhibiting loss of the full-length *bHLH115* transcript (Fig. 1B). When grown vertically on Fe-sufficient medium for 10 d, no visible differences were observed between the wild-type and the two mutants. However, under Fe-deficient conditions the two mutants developed significantly shorter roots compared with the wild-type (Fig. 1C). Further investigation revealed that both the *bhlh115-1* and *bhlh115-2* mutants had lower chlorophyll content compared with the wild-type plants under Fe-deficient conditions, but not under Fe-sufficient conditions (Fig. 1D). Elevation of ferric chelate reductase activity in roots is a characteristic of Fe deficiency in *Arabidopsis thaliana* (Yi and Guerinot, 1996). A ferrozine assay was therefore used to quantify the ferric chelate reductase activity in the roots of wild-type and *bhlh115* mutants. Under Fe-sufficient conditions, the ferric chelate reductase activity of the *bhlh115* mutants was similar to that of the wild-type, whereas under Fe-deficient conditions activity was significantly lower in the mutants (Fig. 1E). To further investigate root responses to Fe deficiency, 4-d-old seedlings grown vertically on Fe-sufficient medium were transferred to Fe-deficient medium for 4 d. Compared with wild-type plants, primary root elongation of the mutants was strongly inhibited by Fe deficiency (Fig. 1F).

### *Overexpression of* bHLH115 *causes increased Fe accumulation*

**Fig. 2.**
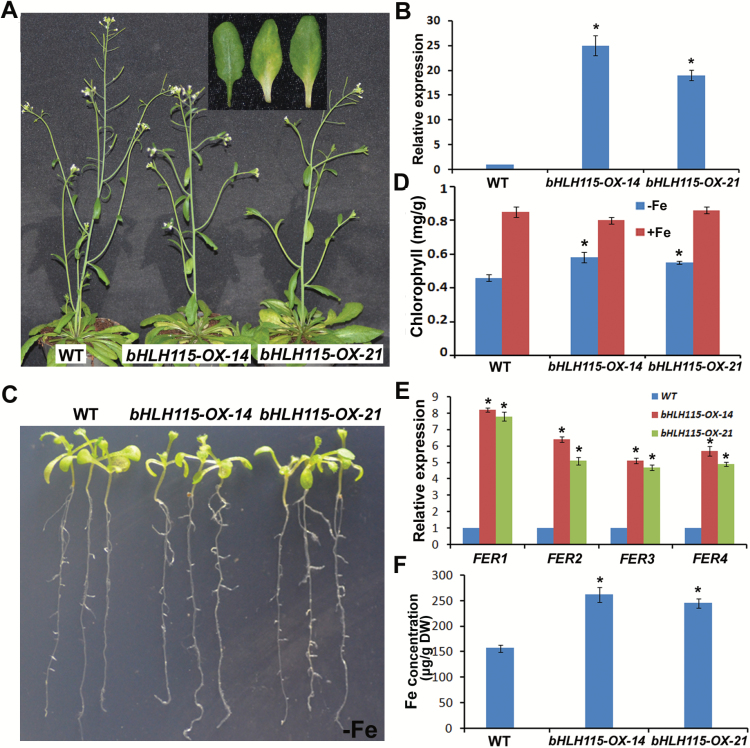
Phenotypes of *bHLH115-OX* transgenic plants. (A) Images of 50-d-old plants that were grown in soil at pH 6.0–6.5. The seventh or eighth leaves are shown in the inset, from left to right: wild-type, *bHLH115-OX-14*, and *bHLH115-OX-21*. (B) Relative expression of *bHLH115* in the transgenic plants. (C) Images of 10-d-old seedlings that were germinated directly on –Fe media. (D) Chlorophyll content of leaves on –Fe or +Fe media. Significant differences from the wild-type were determined by Student’s *t*-test, *P<0.05). (E) Relative expression of *FER* genes in the leaves. Shoots of 10-d-old seedlings germinated directly on +Fe media were used for RNA extraction. Significant differences from the wild-type were determined by Student’s *t*-test, *P<0.05). (F) Fe concentration in the leaves. Shoots of 10-d-old seedlings germinated directly on +Fe media were used for Fe measurement. Significant differences from the wild-type were determined by Student’s *t*-test, *P<0.05).

To further investigate the functions of *bHLH115*, we constructed *bHLH115-OX* transgenic plants in which the CaMV 35S promoter was used to drive expression of a *HA-bHLH115* fusion gene. Forty-nine *bHLH115-OX* transgenic plants were obtained, 40 of which produced chlorotic rosette leaves at the reproductive stage (Fig. 2A). Two independent transgenic plant lines with elevated *bHLH115* levels were used for further analysis (Fig. 2B). Considering the weak Fe-deficiency response of the *bhlh115* mutants, we examined whether *bHLH115-OX* plants had an opposite response. When subjected to Fe-deficient medium, *bHLH115-OX* plant leaves were greener than those of the wild-type (Fig. 2C). Consistent with this, the chlorophyll content of *bHLH115-OX* leaves was higher than in the wild-type (Fig. 2D). However, they developed identical root lengths irrespective of Fe status (Fig. 2C; Supplementary Fig. S1). Having confirmed a bHLH115-regulated plant response to Fe deficiency, we speculated that bHLH115 might affect Fe accumulation in plants. Moreover, ferritins are Fe-storage proteins that sequester or release Fe upon demand (Harrison and Arosio, 1996). There are four *FER* genes encoding ferritins in Arabidopsis, and their expression increases in response to Fe overloading (Petit *et al.*, 2001). Expression analysis indicated that all four *FER* genes were significantly up-regulated in *bHLH115-OX* leaves compared with the wild-type (Fig. 2E). Next, we examined the Fe concentration of 10-d-old shoots grown in Fe-sufficient conditions (Fig. 2F), and found that *bHLH115-OX* shoots had a higher Fe content than the wild-type.

### *Expression of Fe-deficiency-responsive genes is positively correlated with* bHLH115

**Fig. 3.**
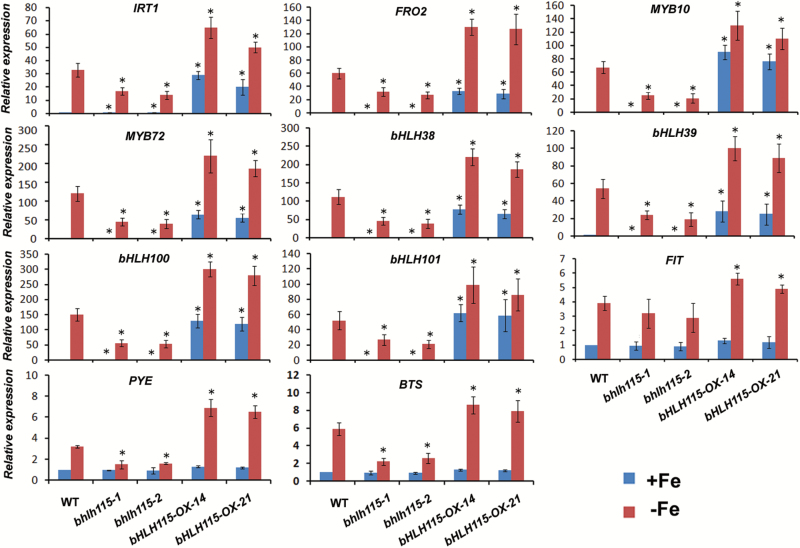
Expression of Fe-deficiency-responsive genes in wild-type, mutants, and overexpression plants. Wild-type, mutants, and transgenic plants were grown on +Fe medium for 4 d, then transferred to +Fe or –Fe media for 3 d. RNA was prepared from root tissues. The data represent means (±SD) of three biological replicates. Significant differences from the wild-type were determined by Student’s *t*-test, *P<0.05).

The above findings suggested that bHLH115 positively regulates the Fe-deficiency response and Fe homeostasis. As a transcription factor, bHLH115 is expected to affect the expression of many downstream genes. We therefore examined whether the susceptibility of *bhlh115* mutants and the tolerance of *bHLH115-OX* plants to Fe deficiency were due to the altered expression of Fe-deficiency-responsive genes. The expression of the Fe uptake-associated genes *IRT1* and *FRO2* increases in response to Fe-deficiency conditions (Robinson *et al.*, 1999; Connolly *et al.*, 2002). Two MYB genes, *MYB10* and *MYB72*, are also induced under Fe-deficient conditions (Palmer *et al.*, 2013). Six bHLH genes, *FIT*, *bHLH38*/*39*/*100*/*101*, and *PYE*, are major regulators of Fe homeostasis and are up-regulated under Fe-deficient conditions (Colangelo and Guerinot, 2004; Wang *et al.*, 2007; Long *et al.*, 2010). Additionally, *BTS* is induced by Fe deficiency and negatively regulates Fe homeostasis (Selote *et al.*, 2015). Under normal growth conditions, the transcript abundance of these Fe-deficiency genes was low in *bhlh115* mutants and high in *bHLH115-OX* plants compared with the wild-type (Fig. 3). Although Fe-deficiency-responsive genes were induced by Fe deficiency in both *bhlh115* mutants and *bHLH115-OX* plants, expression levels were lower in *bhlh115* mutants and higher in *bHLH115-OX* plants than observed in the wild-type. This suggests that the expression of Fe-deficiency-responsive genes is positively correlated with *bHLH115* expression.

### *bHLH115 directly activates the transcription of* bHLH38/39/100/101 *and* PYE

**Fig. 4.**
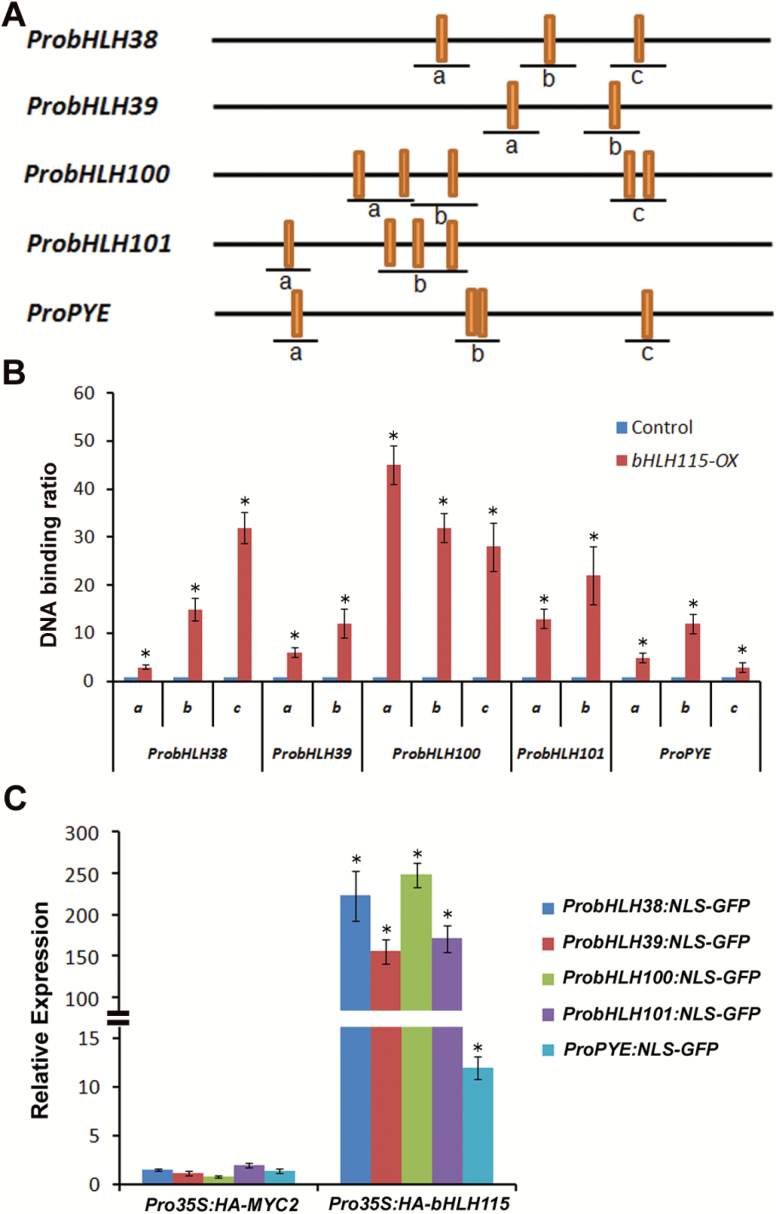
Direct regulation of *bHLH38*/*39*/*100*/*101* and *PYE* by bHLH115. (A) E-box in promoters. The bars indicate the E-box (CANNTG). The 1000-bp upstream sequence from the translation start site of the indicated gene is shown. (B) ChIP-qPCR analyses of the DNA binding ratio of bHLH115 to the promoters of *bHLH38*/*39*/*100*/*101* and *PYE* genes. qPCR was used to quantify enrichment of the indicated promoters and a fragment of the *TUB2* promoter containing an E-box motif was used as a negative control. The binding to the *TUB2* promoter fragment in the wild-type was set to 1 and used to normalize the DNA binding ratio. Significant differences were determined by Student’s *t*-test, *P<0.05. (C) bHLH115 activates the promoters of *bHLH38*/*39*/*100*/*101* and *PYE*. In the transient assays, *Pro35S:Myc-GUS* was expressed as an internal control. *GFP* transcript abundance was normalized to the *GUS* transcript. The value obtained with the empty vector as an effector was set to 1. The results are means (±SD) of three biological replicates. Significant differences were determined by Student’s *t*-test, *P<0.05.

As bHLH115 is a close homologue of bHLH34, bHLH104, and bHLH105, which directly activate transcription of *bHLH38*/*39*/*100*/*101* and *PYE* (Zhang *et al.*, 2015; Li *et al.*, 2016), we hypothesized that bHLH115 could also directly activate *bHLH38*/*39*/*100*/*101* and *PYE*. To test this hypothesis, ChIP-PCR experiments were conducted using *bHLH115-OX* transgenic roots grown under Fe-deficient conditions. These assays revealed that promoter sequences of *bHLH38*/*39*/*100*/*101* and *PYE* spanning E-box motifs (CAXXTG) (Fig. 4A) were highly enriched in anti-HA-immunoprecipitated chromatin in *bHLH115-OX* transgenic plants but not in wild-type plants, thus demonstrating direct binding of bHLH115 to the promoters of *bHLH38*/*39*/*100*/*101* and *PYE* (Fig. 4B). Transient expression assays were performed to investigate whether bHLH115 activated the above promoters. *Pro35S:HA-bHLH115* was used as an effector and *ProbHLH38:NLS-GFP* as a reporter. Co-expression of *Pro35S:HA-MYC2* and *ProbHLH38:NLS-GFP* resulted in GFP transcript levels similar to that in the co-expression of the empty vector and *ProbHLH38:NLS-GFP*, whereas the co-expression of *Pro35S:HA-bHLH115* and *ProbHLH38:NLS-GFP* caused high levels of GFP (Fig. 4C). These data suggest that bHLH115 activates the *bHLH38* promoter. Using a similar approach, it was confirmed that bHLH115 could also activate the *bHLH39*, *bHLH100*, *bHLH101*, and *PYE* promoters.

### bHLH34/104/105/115 share similar molecular functions

**Fig. 5.**
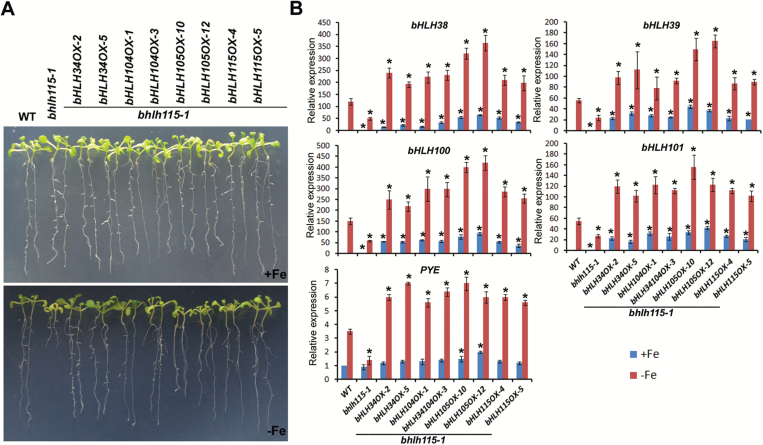
Complementation of *bhlh115-1* by *bHLH34*/*104*/*105*/*115*. (A) Images of 10-d-old seedlings that were germinated directly on +Fe or –Fe media. (B) Plants were grown on +Fe media for 4 d, then transferred to +Fe or –Fe media for 3 d. RNA was prepared from root tissues. The data represent means (±SD) of three biological replicates. Significant differences from the wild-type were determined by Student’s *t*-test, *P<0.05).

Our results and previous studies show that all four members of the bHLH subgroup IVc can activate expression of *bHLH38*/*39*/*100*/*101* and *PYE*, suggesting their redundant molecular functions. Our previous work confirmed that overexpression of *bHLH101* only rescued Fe-deficiency induction of *IRT1* and *FRO2*, but not of *MYB10* and *MYB72,* in the *bhlh34 bhlh104* double-mutant (Li *et al.*, 2016). This suggests that unknown target genes of bHLH34 and bHLH104 have yet to be identified. It remains unclear whether the four proteins share the same, as yet unidentified, target genes or have identical regulatory functions. To explore this hypothesis, each of the four genes was driven by the CaMV 35S promoter and separately introduced into the *bhlh115-1* mutant plants. Fe-deficiency assays indicated that the overexpression of each gene allowed for complete rescue of Fe-deficiency sensitivity of the *bhlh115-1* mutant plants under Fe-deficiency conditions (Fig. 5A). An examination of Fe-deficiency-responsive genes indicated that the expression of *bHLH38*/*39*/*100*/*101* and *PYE* in *bhlh115-1* was completely rescued by the overexpression of bHLH subgroup IVc members. These data suggest similar molecular functions among bHLH34/104/105/115.

### Physiological interactions between bHLH115 and bHLH34/104/105/115

**Fig. 6.**
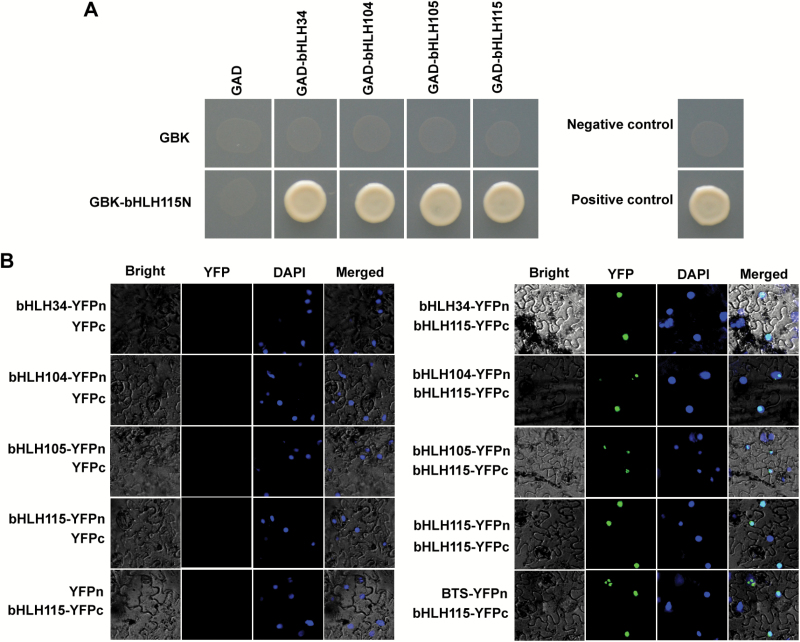
Interaction between bHLH115 and bHLH34/104/105/115. (A) Yeast two-hybrid assays. Interaction was indicated by the ability of cells to grow on synthetic dropout (SD) medium lacking Leu/Trp/His/Ade. N-terminal truncated *bHLH115* was cloned into pGBKT7, and full-length cDNAs of *bHLH34*, *bHLH104*, *bHLH105*, and *bHLH115* were cloned into pGADT7. (B) BiFC assays Of *N. benthamiana* leaves infiltrated with different combinations of the indicated constructs.

bHLH transcription factors can function as homodimers or heterodimers (Toledo-Ortiz *et al.*, 2003). Our previous study confirmed formation of homodimers or heterodimers between bHLH34, bHLH104, and bHLH105 (Li *et al.*, 2016). Given their similar molecular functions, we examined whether all members of bHLH subgroup IVc could interact with themselves or other members. Yeast-two-hybrid (Y2H) assays were used to test this hypothesis. Owing to the strong autoactivation caused by full-length bHLH115 fused with the BD domain (see Supplementary Fig. S2), a truncated N-terminal bHLH115 (bHLH115N) with no autoactivation was used. Full-length bHLH subgroup IVc members were fused with the AD domain. In yeasts grown on SD medium without Leu/Trp/His/Ade, bHLH115 was found to interact with each member of the bHLH subgroup IVc (Fig. 6A).

Bimolecular fluorescent complementation (BiFC) assays were conducted to further confirm these interactions. Full-length bHLH115 was fused with the C-terminal of YFP (bHLH115-cYFP), and full-length bHLH34/104/105/115 were fused with the N-terminal of YFP (bHLH34-nYFP, bHLH104-nYFP, bHLH105-nYFP, and bHLH115-nYFP), respectively. Transient co-expression assays were conducted in *N. benthamiana* leaf cells. Strong YFP signals were observed in combinations of bHLH115-cYFP with bHLH34-nYFP, bHLH104-nYFP, bHLH105-nYFP, and bHLH115-nYFP, as well as the positive control BTS-nYFP, with no GFP signals detected in the negative controls (Fig. 6B); this was in agreement with the (Y2H) assay results (Fig. 6A). The fluorescence signals co-localized with the DAPI signal from the nuclei, and this is consistent with activity of transcription factors in the nuclei.

### Genetic interactions between bHLH115 and bHLH34/104/105

**Fig. 7.**
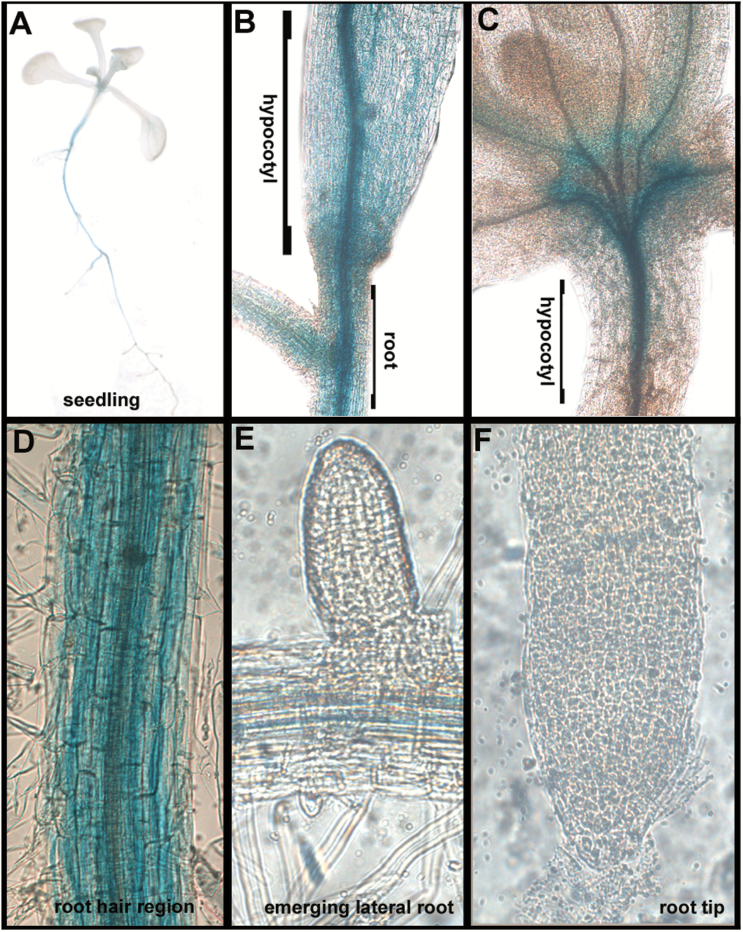
Expression of a *ProbHLH115:GUS* fusion. Images of 10-d-old seedlings that were germinated directly on +Fe medium and used for GUS staining. (A) Seedling, (B) hypocotyl and root, (C) connected region between petioles and hypocotyl, (D) root hair zone, (E) emerging lateral root, and (F) root tip.

Having confirmed that bHLH34, bHLH104, bHLH105, and bHLH115 have similar regulatory functions, we wanted to examine whether bHLH115 had a specific role in specific tissues or organs. Firstly, expression of *bHLH115* was determined after Fe-deficiency treatment, suggesting that its transcription was independent of Fe status (see Supplementary Fig. S3). Then, *ProbHLH115:GUS* transgenic plants were constructed, in which the *GUS* reporter gene was driven by the *bHLH115* promoter. GUS staining suggested that *ProbHLH115:GUS* was present in roots, hypocotyls, and shoot meristems (Fig. 7). Further examination of root tissues revealed that *ProbHLH115:GUS* was expressed in root maturation regions, but not in initial lateral roots and root tips. The expression pattern of *bHLH115* was different from that observed for *bHLH34*, *bHLH104*, and *bHLH105* (Li *et al.*, 2016), implying that bHLH115 may play non-redundant biological functions.

**Fig. 8.**
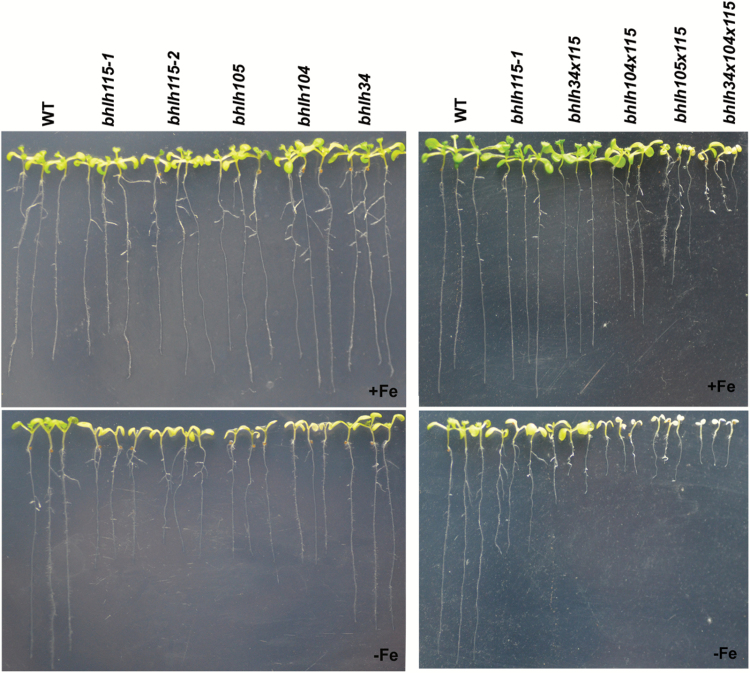
Phenotypes of various mutants. Images of 10-d-old seedlings germinated directly on +Fe or –Fe media.

Subsequently, Fe-deficiency phenotypes of *bhlh115* mutants were compared with *bhlh34*, *bhlh104*, and *bhlh105* mutants. This revealed that the Fe-deficiency symptoms of the *bhlh115* mutants were similar to those of *bhlh104* and *bhlh105*, but more severe than those of *bhlh34* (Fig. 8; Supplementary Fig. S4). This suggests that each of these genes may have an independent biological function. Next, three double-mutants (*bhlh34 × 115*, *bhlh104 × 115*, and *bhlh105 × 115*) and one triple-mutant (*bhlh34 × 104 × 115*) were constructed by crossing the *bhlh115-1* mutant with *bhlh34*, *bhlh104*, or *bhlh105* (Fig. 8). We failed to obtain other triple-mutants and quadruple-mutants. When grown on Fe-sufficient media, in comparison with the *bhlh115-1* single-mutant, all multiple mutants had short roots, with triple-mutant plants having the shortest; *bhlh105 × 115* and *bhlh34 × 104 × 115* developed severely chlorotic leaves, and exhibited delayed growth. When grown in Fe-deficient media, *bhlh104 × 115*, *bhlh105 × 115*, and *bhlh34 × 104 × 115* plants developed albino cotyledons and their shoots ceased growing before the emergence of true leaves. To further investigate root elongation in the Fe-deficient medium, 4-d-old seedlings grown in Fe-sufficient medium were transferred to Fe-deficient medium for 3d. As shown in Supplementary Fig. S5, root elongation of the various mutants was inhibited by Fe deficiency. When grown in soil, *bhlh115-1*, *bhlh34 × 115*, and *bhlh104 × 115* developed normally, as did wild-type plants; in contrast, *bhlh105 × 115* and *bhlh34 × 104 × 115* plants could not survive unless exogenous Fe was supplied. Even with exogenous Fe application, the growth and development of *bhlh105 × 115* and *bhlh34 × 104 × 115* plants were severely retarded (see Supplementary Fig. S6). Taken together, bHLH115 has independent roles and also plays an additive role with bHLH34, bHLH104, and bHLH105 in Fe homeostasis.

### Genetic interaction between bHLH115 and BTS

**Fig. 9.**
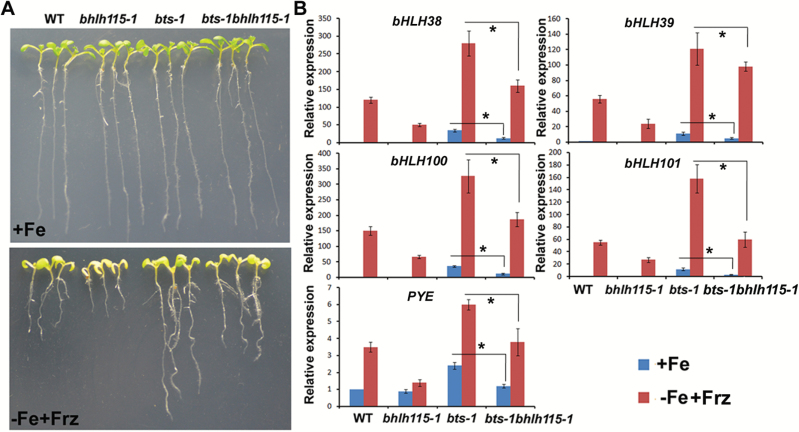
Negative regulation of bHLH115 by BTS. (A) Images of 10-day-old plants that were grown under either Fe-sufficient or Fe-free plus Frz (50 uM) conditions. (B) Expression of Fe-deficiency-responsive genes. Plants were grown on +Fe media for 4 d, then transferred to Fe-sufficient or Fe-free plus Frz media for 3 d. RNA was prepared from root tissues. Significant differences between two samples were determined by Student’s *t*-test, *P<0.05.

A previous study revealed that BTS interacts with bHLH115 and facilitates 26S proteasome-mediated degradation of bHLH115 proteins in the absence of Fe (Selote *et al.*, 2015). To investigate the genetic interaction between bHLH115 and BTS, the *bts-1 bhlh115-1* double-mutant was constructed by crossing *bts-1* and *bhlh115-1*. No visible phenotype differences were observed among *bhlh115-1*, *bts-1*, and *bts-1 bhlh115-1* under Fe-sufficient conditions. Another previous study confirmed that *bts-1* shows enhanced Fe-deficiency tolerance under Fe-free plus ferrozine (Frz) conditions (Long *et al.*, 2010). Consequently, we examined the Fe-deficiency tolerance of various mutants under Fe-free plus Frz conditions. The *bts-1* mutant developed the longest roots, whereas *bts-1 bhlh115-1* produced significantly shorter roots compared with *bts-1* (Fig. 9A), suggesting that loss-of-function of *bHLH115* compromised the Fe-deficiency tolerance of *bts-1*. We wanted to know whether the reduced Fe-deficiency tolerance of *bts-1 bhlh115-1* compared with *bts-1* resulted from the disrupted expression of Fe-deficiency-responsive genes, and therefore we examined the expression of these genes. Consistent with the Fe-deficiency tolerance of *bts-1*, the expression of *bHLH38*/*39*/*100*/*101* and *PYE* was dramatically up-regulated under Fe-sufficient or Fe-free plus Frz conditions. Despite the up-regulation of *bHLH38*/*39*/*100*/*101* and *PYE* in the *bts-1 bhlh115-1* mutant, their expression in the *bts-1 bhlh115-1* double-mutant was significantly lower than in the *bts-1* mutant (Fig. 9B).

## Discussion

Iron plays important roles in the regulation of many biochemical and physiological processes. When plants are exposed to Fe-deficient conditions, they can activate their Fe acquisition system for uptake. Plants have evolved a series of strategies to meet Fe demands, such as facilitating acquisition and remobilizing internal Fe. The intracellular concentration of Fe in plants is tightly controlled through the regulation of many transcription factors. For example, FIT and bHLH38/39/100/101 interact physiologically and positively regulate expression of the strategy-I associated genes *IRT1* and *FRO2* (Yuan *et al.*, 2008; Wang *et al.*, 2013). MYB10 and MYB72 directly activate *NAS4* under Fe-deficient conditions (Palmer *et al.*, 2013). PYE plays a key role in maintaining Fe homeostasis by negatively regulating *ZIF1*, *FRO3*, and *NAS4* (Long *et al.*, 2010). Previous studies showed that bHLH34/104/105 maintain Fe homeostasis by positively regulating *bHLH38*/*39*/*100*/*101* and *PYE* (Zhang *et al.*, 2015; Li *et al.*, 2016). Here, we show that bHLH115 is a novel regulator of *bHLH38*/*39*/*100*/*101* and *PYE*.

### *bHLH115 targets* bHLH38/39/100/101 *and* PYE

bHLH115 is a member of the bHLH IVc subgroup. In this study, loss-of-function of *bHLH115* caused a weak Fe-deficiency response, with plants displaying shorter roots under Fe-deficiency conditions (Fig. 1C). In contrast, overexpression of *bHLH115* caused Fe over-accumulation (Fig. 2F). Thus, appropriate expression of *bHLH115* is required for maintenance of Fe homeostasis. Several Fe-deficient mutants (*frd1*, *man*, *irt1*, and *fit*) display enhanced expression of *bHLH38*/*39*/*100*/*101* under both Fe-sufficient and Fe-deficient conditions (Wang *et al.*, 2007). In contrast, their expression was down-regulated in *bhlh115* mutants. This suggests that *bHLH38*/*39*/*100*/*101* function downstream of *bHLH115*. ChIP assays confirmed that bHLH115 binds to the promoters of *bHLH38/39/100/101* and *PYE* (Fig. 4B). Transient expression assays indicated that bHLH115 positively regulates expression of *bHLH38*/*39*/*100*/*101* and *PYE* (Fig. 4C). As target genes of bHLH115, *bHLH38*/*39*/*100*/*101* were all up-regulated in *bHLH115-OX* plants (Fig. 3). Moreover, *FIT* expression is up-regulated in *bHLH115-OX* plants (Fig. 3). In agreement that FIT and bHLH38/39/100/101 synergistically regulate Fe uptake, expression of the Fe uptake-associated genes *IRT1* and *FRO2* was also activated in *bHLH115-OX* plants (Fig. 3), thus explaining the elevated Fe accumulation in these plants (Fig. 2F). Recent studies have confirmed that homologs of bHLH115 (bHLH34/104/105) positively regulate expression of *bHLH38*/*39*/*100*/*101* and *PYE* (Zhang *et al.*, 2015; Li *et al.*, 2016). However, our results suggested that the single-mutant of *bHLH115* could cause a disrupted Fe-deficiency response, indicating that *bHLH115* is required for the normal Fe-deficiency response. When the *bhlh115* mutant was crossed with *bhlh34*, *bhlh104*, and *bhlh105* mutants, each double-mutant displayed more severe Fe-deficiency symptoms than the single-mutant (Fig. 8).

### Functional similarity and differences among bHLH IVc subgroup members

The bHLH IVc subgroup contains four members, bHLH34/104/105/115, three of which, bHLH34/104/105, have been confirmed to positively regulate Fe homeostasis in *Arabidopsis thaliana* (Zhang *et al.*, 2015; Li *et al.*, 2016). Under both Fe-sufficient and Fe-deficient conditions, expression of *bHLH38*/*39*/*100*/*101* and *PYE* in the *bhlh115* mutants was lower than observed in wild-type plants; however, their expression was still induced by Fe limitation in *bhlh115* mutants, implying that other members of the bHLH IVc subgroup may activate their expression under Fe-deficient conditions. Expression analysis confirmed that Fe-deficiency-induction of *bHLH38*/*39*/*100*/*101* and *PYE* was dramatically weakened in the *bhlh34 × 115*, *bhlh104 × 115*, *bhlh105 × 115*, and *bhlh34 × 104 × 115* mutants compared with the *bhlh115-1* mutant (see Supplementary Fig. S7), indicating that bHLH34/104/105 are responsible for induction of *bHLH38*/*39*/*100*/*101* and *PYE* under Fe-deficient conditions in the *bhlh115-1* mutant. Thus, each member of the bHLH IVc subgroup shares similar molecular functions, namely being able to activate expression of *bHLH38*/*39*/*100*/*101* and *PYE*. Genetic complementation assays demonstrated that each of the bHLH IVc subgroup members could rescue the *bhlh115-1* mutant under Fe-deficient conditions, thus confirming similar regulatory functions.

Loss-of-function of anyone of *bHLH34*/*104*/*105*/*115* can result in an abnormal Fe-deficiency response, with double- and triple-mutants displaying more severe Fe-deficiency symptoms compared with single-mutants (Fig. 8; Zhang *et al.*, 2015; Li *et al.*, 2016). Comparative analysis revealed their expression patterns are different (Fig. 8; Li *et al.*, 2016). In the root maturation zone, *bHLH34*/*104*/*105* were expressed in the endodermis and pericycle, whereas *bHLH115* was expressed in all tissues. *bHLH34*/*104*, but not *bHLH105*/*115*, were expressed in the initial lateral roots. *bHLH105*was detected in the whole root tip, *bHLH34*/*104* were expressed in the meristem of the root tip, whereas *bHLH115* was not detected in the root tip. *bHLH105* was not expressed in the hypocotyl, but *bHLH34*/*104*/*115* were. In addition, *bHLH115* was the only gene not expressed in the leaf veins, but was ubiquitously expressed in the root mature zone (Fig. 7), implying putative specific roles for bHLH115 in Fe uptake. These differential expression patterns imply specific biological functions in specific tissues. Protein interaction assays indicated potential homo- or heterodimerization among bHLH IVc subgroup members. Furthermore, overlapping expression patterns in the root, hypocotyl, and leaf were observed (Fig. 7; Li *et al.*, 2016). This suggests they may function synergistically to maintain Fe homeostasis in specific tissues.

Recently, the chrysanthemum and apple homologs of the bHLH IVc subgroup members, CmbHLH1 and MdbHLH104, were found to positively regulate Fe accumulation by activating transcription of the gene encoding H^+^-ATPase (Zhao *et al.*, 2014, 2016), suggesting conserved functions in the regulation of Fe homeostasis. We did not observed visible changes in GUS-staining in the *ProbHLH115:GUS* plants after Fe-deficiency treatment. Expression analysis confirmed that the *bHLH115* transcript was not responsive to Fe deficiency (see Supplementary Fig. S3). Similarly, the expression of *bHLH34*/*104*/*105* is not affected by Fe deficiency (Zhang *et al.*, 2015; Li *et al.*, 2016). Unlike Arabidopsis bHLH IVc subgroup members, transcript abundance of both *CmbHLH1* and *MdbHLH104* increases under Fe-deficiency conditions, indicating evolutional divergence among different plants species.

### Negative regulation of bHLH IVc subgroup members by BTS

BTS is a negative regulator of Fe homeostasis, and its mutant accumulates more Fe than wild-types under Fe-sufficient conditions (Selote *et al.*, 2015; Zhang *et al.*, 2015). bHLH105 and bHLH115 physiologically interact with BTS *in vivo* and can be degraded by BTS *in vitro* (Selote *et al.*, 2015). Zhang *et al.* (2015) demonstrated that elevated expression of *bHLH105* can promote Fe accumulation in plants, and this work showed that *bHLH115* had a similar function; this may explain the Fe over-accumulation observed in plants with loss-of-function of *BTS*. Our genetic analysis was in agreement this, revealing that the loss-of-function of *bHLH115* compromised the Fe-deficiency tolerance of the *bts-1* mutant. Corresponding with this, the expression of *bHLH38*/*39*/*100*/*101* and *PYE* in *bts-1 bhlh115-1* plants was lower than in *bts-1* plants. These data suggest that bHLH115 functions downstream of BTS. Despite its interaction with BTS, the bHLH104 protein is not degraded by BTS *in vitro* (Selote *et al.*, 2015). Likewise, loss-of-function of *bHLH104* also alleviates Fe-deficiency tolerance of the *bts-1* mutant (Zhang *et al.*, 2015), implying a potential negative regulation of bHLH104 by BTS. However, the mechanism by which BTS affects bHLH104 functions remains unclear.

To maintain Fe concentrations within physiological limits, plants modulate processes contributing to Fe homeostasis in accordance with the external supply and internal requirements. BTS was identified as a potential Fe sensor. Both its transcripts and proteins are up-regulated under Fe-deficient conditions; however, its proteins decrease upon Fe treatment (Selote *et al.*, 2015). It has been proposed that under Fe-deficient conditions the elevated levels of BTS facilitate degradation of bHLH105 and bHLH115, resulting in down-regulation of Fe-deficiency-responsive genes. However, this inference is not consistent with the up-regulation of Fe-deficiency-responsive genes under Fe-deficiency conditions. Therefore, other factors are likely to activate bHLH105 and bHLH115 functions by antagonizing BTS under Fe-deficiency conditions. Further investigation is required to explain this paradox.

Based on our findings and those of previous studies, we propose a working model for bHLH115 and its homologues. Under Fe-deficiency conditions, IVc subgroup bHLH transcription factors bHLH34/104/105/115 activate the expression of *PYE*, *FIT*, and *bHLH38*/*39*/*100*/*101*. FIT and bHLH38/39/100/101 synergistically activate the expression of the Fe-uptake-associated genes *IRT1* and *FRO2*. PYE directly regulates the expression of the Fe distribution-associated genes *ZIF1* and *NAS4*. bHLH34/104/105/115 play synergistic roles in the overlapping expression locations and each has independent roles in their specific expression locations.

## Author contributions

GL and DY designed the study; GL, HZ, XL, and QA performed the research; GL, HZ, XL, and DY analysed the data. GL wrote the paper; GL, HZ, XL, and DY revised the paper. All authors read and approved the final manuscript.

## Supplementary Material

Supplementary Data
