# Supplementary material for: bHLH transcription factor bHLH115 regulates iron homeostasis in Arabidopsis thaliana
Source: J Exp Bot. 2017 Mar 28;68(7):1743–55. doi: 10.1093/jxb/erx043 (PMC5441899; doi:10.1093/jxb/erx043)
Supplement: Supplementary Data [file erx043_Supplementary_Data.zip › supplementary_figures_S1_S7_Table_S1.pdf]

bHLH transcription factor bHLH115 regulates iron homeostasis in *Arabidopsis thaliana*

Gang Liang[1], Huimin Zhang[1,3], Xiaoli Li[1,3], Qin Ai[1,2], Diqiu Yu[1]*

[1]Key Laboratory of Tropical Plant Resources and Sustainable Use, Xishuangbanna Tropical Botanical Garden, Chinese Academy of Sciences Kunming, Yunnan 650223, China

[2]University of Chinese Academy of Sciences, Beijing 100049, China

[3]School of Life Sciences, University of Science and Technology of China, Hefei, Anhui 230027, China

***Corresponding author:**

Diqiu Yu (ydq@xtbg.ac.cn)

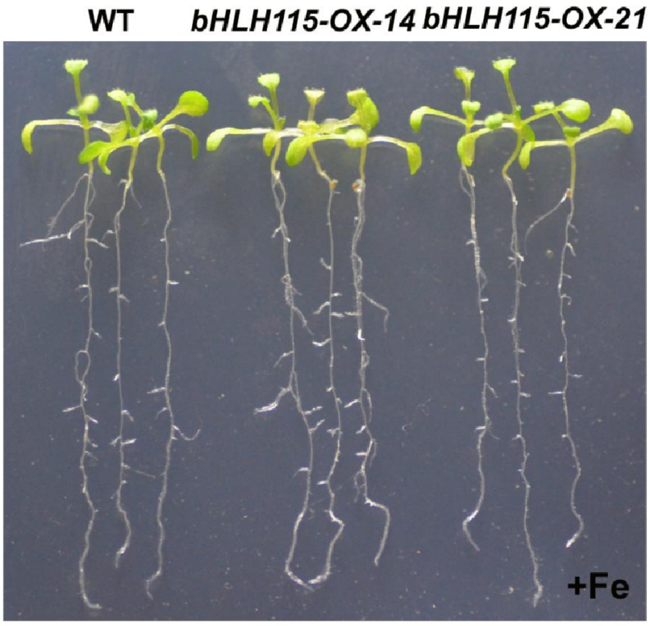

Figure S1. ***bHLH115-OX*** **plants grown on +Fe media.**

10-day-old seedlings were shown.

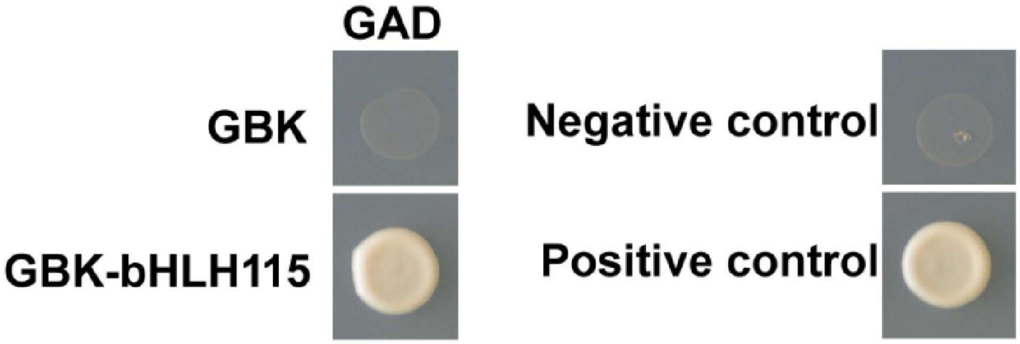

Figure S2. bHLH115 shows autoactivity in yeast.
Yeast two-hybrid assays. Interaction was indicated by the ability of cells to grow on synthetic dropout medium lacking Leu/Trp/His/Ade. Full-length *bHLH115* was cloned into pGBKT7.

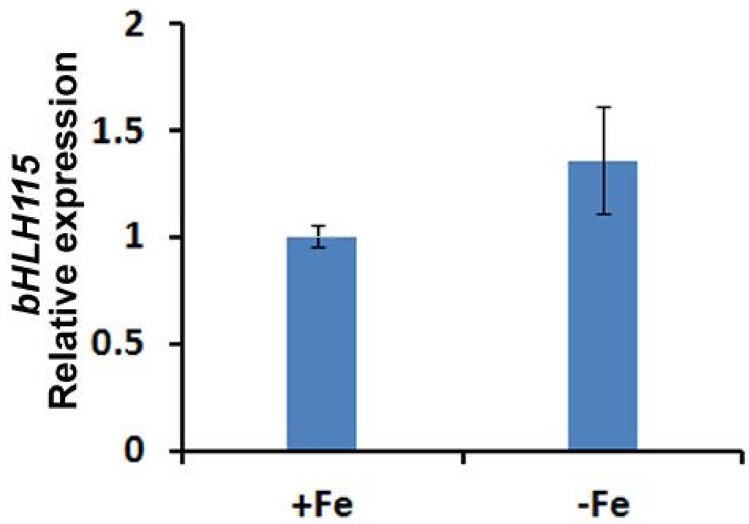

Figure S3. Expression of *bHLH115* in response to Fe deficiency.

Plants were grown on +Fe media for 4 days, then transferred to +Fe or -Fe media for 3 days. RNA was prepared from root tissues. The data represent means (±SD) of three biological replicates.

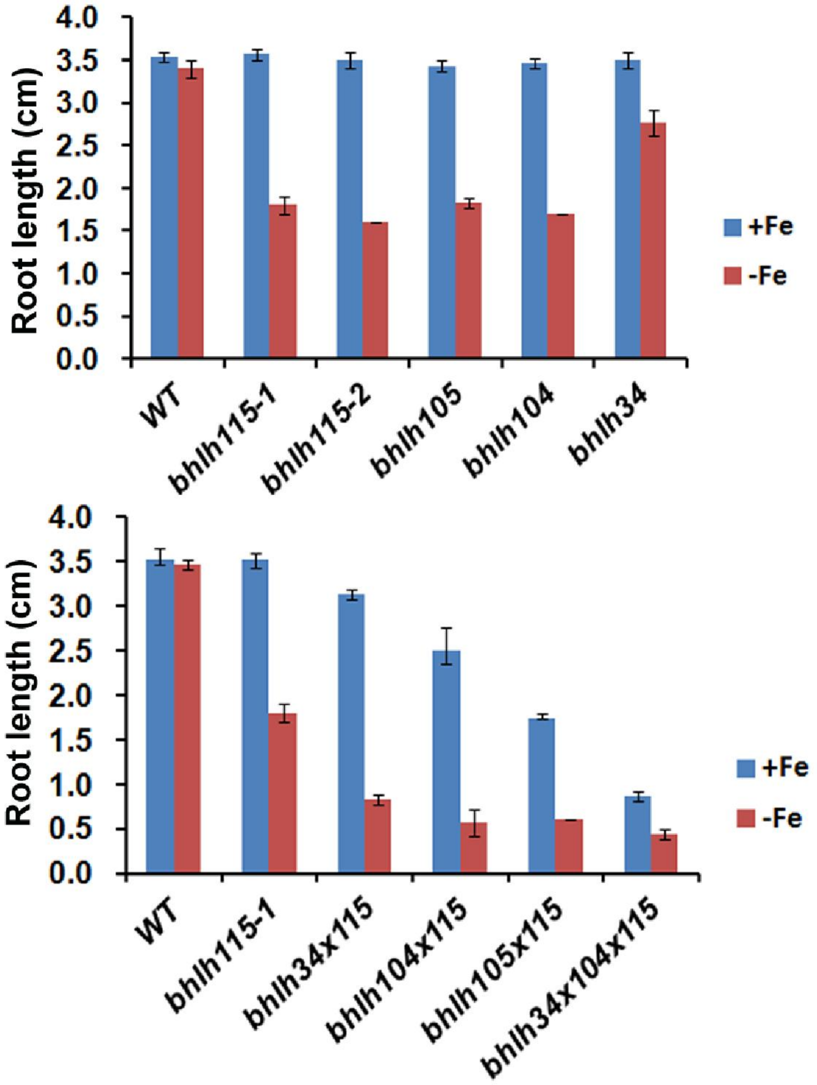

Figure S4. The root length of various mutant plants.
10-day-old seedlings germinated directly on +Fe or -Fe media.

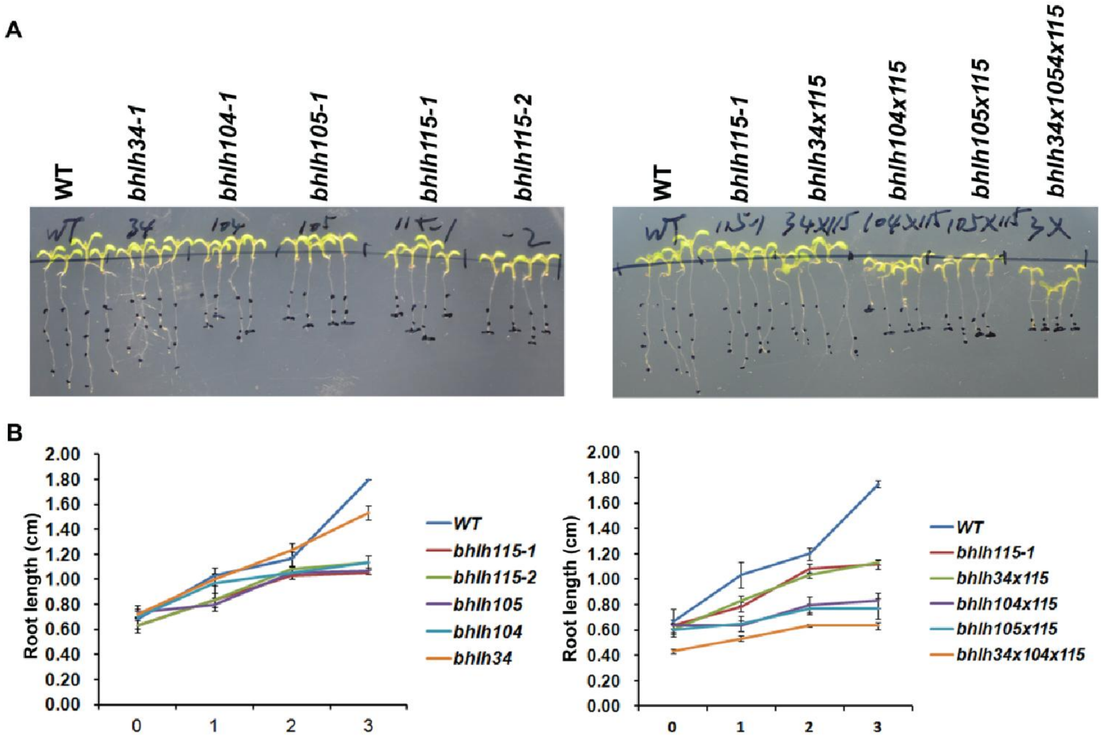

Figure S5. Root elongation of various mutants under Fe deficient conditions.

(A) Representative figures were shown. The 1st, 2nd, 3rd and 4th dots (from top to bottom) indicate the position of root tip 0, 1, 2 and 3 days after transfer to –Fe media, respectively. The dash indicates the overlapping 3rd and 4th dots.

(B) Time course quantification of root length of plants o to 3 days after transfer to -Fe media.

(A) and (B) 4-day-old seedlings grown on +Fe media were shifted to –Fe media in the root elongation assay.

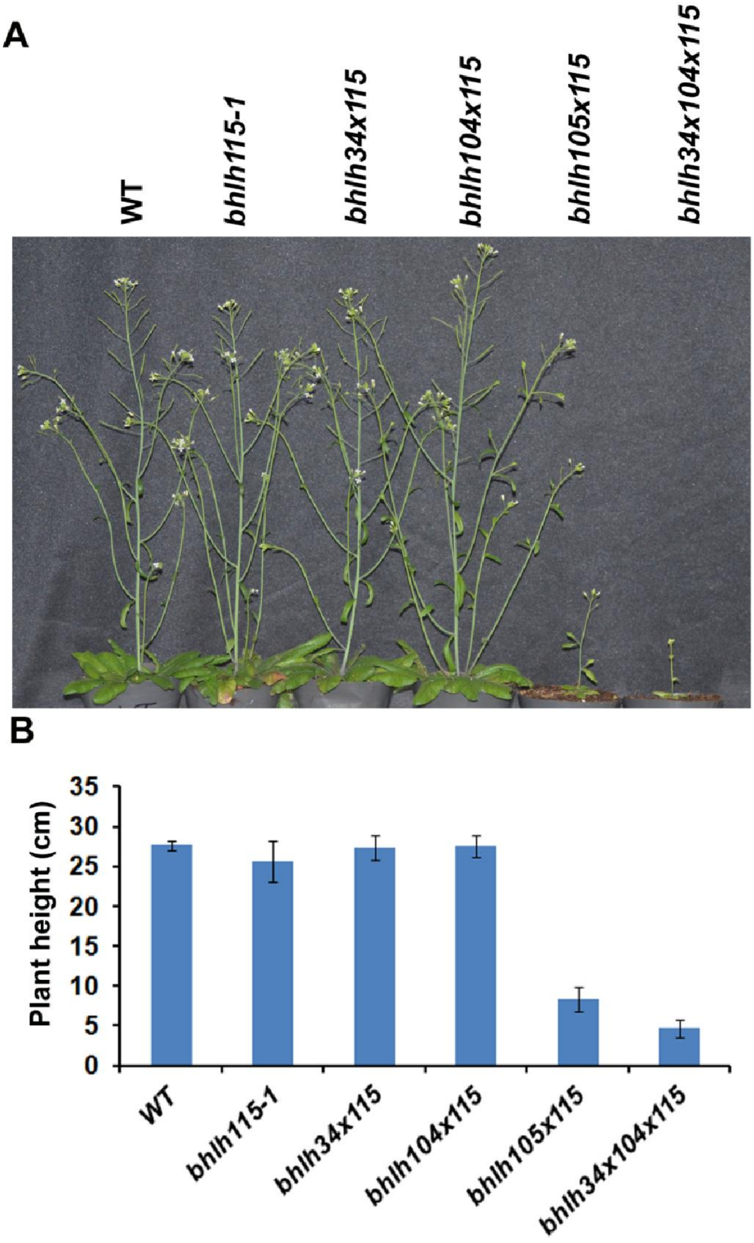

Figure S6. Various mutant plants grown in soils.

(A) Plants were germinated on Fe sufficient media for 7 days and transferred to soils (pH 6.0-6.5). The soils of *bhlh105x115* and *bhlh34x104x115* were supplied with 0.5mM FeEDTA every 5 days.

(B) Plant height of plants in (A).

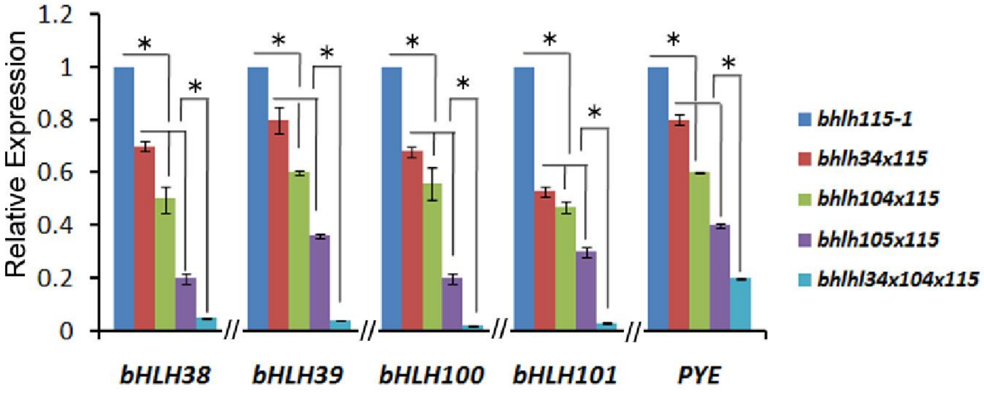

Figure S7. Expression of *bHLH38/39/100/101* and *PYE* in various mutants. Plants were grown on +Fe media for 4 days, then transferred to -Fe media for 3 days. RNA was prepared from root tissues. Significant differences between two samples are indicated by an asterisk ($P < 0.05$), as determined by Student' *t* test.

Supplemental Table 1. Primers used in this study.

**Mutant screening**	
bhlh115-1-A	ATGGTGCTTTGTCAATAGGTTTACG
bhlh115-1-B	TAAGTTGCAGTGGACTATGTCACCA
bhlh115-2-A	GGAGAATGGTGTCTCCGGAGA
bhlh115-2-B	CGCAGGTTTGACCAAATAGCG
P745	AACGTCCGCAATGTGTTATTAAGTTGTC
**Promoter**	
ProbHLH115-F	TTTAAGCTTAGCGGGAACAAAACCTTGGGGA
ProbHLH115-R	AATGTCGACTCTCCTGATTCCCCGGCGGC
**Overexpression**	
bHLH105-F	TTTGAGCTCATGGAGTACCCATACGACGTACCA
bHLH105-R	TTTGTCGACAGCAACAGGAGGACGAAGGAC
bHLH115-F	TTTGAGCTCATGGAGTACCCATACGACGTACCA
bHLH115-R	TTAGGATCCTTAAGCAACTGGAGGACGAAGG
**BiFC**	
YN-bHLH34-F	ATAATTTAAATATGTATCCATCAATCGAAGACGATG
YN-bHLH34-R	ATAGGATCCAGCAACAGGAGGAAGATTTTTG
YN-bHLH104-F	AAGGATCCATGTATCCTTCTCTCGACGATGATT
YN-bHLH104-R	AATCTAGAAGCAGCAGGAGGCCTGAGTT
YN-bHLH105-F	TTTCTAGAATGGTGTCACCCGAAAACGC
YN-bHLH105-R	TTTCTAGAAGCAACAGGAGGACGAAGGA
YN-bHLH115-F	TTGGATCCATGGTGTCTCCGGAGAATACG
YN-bHLH115-R	TTTCTAGAAGCAACTGGAGGACGAAGGAC
YC-bHLH115-F	TTTCTAGAATGGTGTCTCCGGAGAATACG
YC-bHLH115-R	TTGGATCCAGCAACTGGAGGACGAAGGAC
**Yeast**	
BD-bHLH115-F	TTTGAATTCATGGTGTCTCCGGAGAATACGA
BD-bHLH115-N-R	AAGGATCCTACTGTTTCTCATCACGCAGCTC
AD-bHLH115-F	TTTGAATTCATGGTGTCTCCGGAGAATACGA
AD-bHLH115-R	TTAGGATCCTTAAGCAACTGGAGGACGAAGG
**qRT-PCR**	
qBTS-F	AAGGGCCGTTTACCACTGTC
qBTS-R	CTTCTCGAGGCACTTGTGGT
**qPCR for ChIP**	
qbHLH38-a-F	CGAATGTTGGAAACTTCATTGATTC
qbHLH38-a-R	TTTTAATTCCACAATGACGATGGTC
qbHLH38-b-F	ACAACATAAAAAATGTATGGGACGA
qbHLH38-b-R	TTGTGTAAGAATCAAACATGTATAT
qbHLH38-c-F	CATGTTTGATTCTTACACAATATGC
qbHLH38-c-R	TCGTTGAGATTATATGATTGTGTTAT
qbHLH39-a-F	GGAGGTCAACAAATAAATAAAATGC
qbHLH39-a-R	ACATATTGAAAGATGACTCAGCCTG
qbHLH39-b-F	CCAGTCTACTTGTGACTAGACCTTG

qbHLH39-b-R	AACCAAAACTTTAAAAATTCGCAAA
qbHLH100-a-F	AAAATAAACGATAGACACTACACCA
qbHLH100-a-R	ACTATTGAGAGGCACGAGCAAAT
qbHLH100-b-F	ATTTGCTCGTGCCTCTCAATAG
qbHLH100-b-R	GAATATGCAATCACATTTTTCTACC
qbHLH100-c-F	AAGAACATTAGGATATTAATGCCTG
qbHLH100-c-R	TAAATTAAAATACATTGGGTCACGA
qbHLH101-a-F	TTGATAGTGGATCATTAATTACTTGTA
qbHLH101-a-R	ATTTACCATTCAGAGATCCATCC
qbHLH101-b-F	ACAGCAAACATAAAACTTCATGTGG
qbHLH101-b-R	GTTATATTTTGAACATGTGAACGCA
qPYE-a-F	GTTTGTGGGAAACACACGACAG
qPYE-a-R	AGATGAAAAGTAACATTTTCACAAAA
qPYE-b-F	GAGATGAGCTTTAGTGGCACGC
qPYE-b-R	GAAGGTCCGAAGTTGAGGAGGG
qPYE-c-F	CTGTTGTGTTGAGAACTTAGCCGTA
qPYE-c-R	ACTTTCGTGTGATTTTTGATTCCTC
qTUB2-F	TTTCGCTTTCTTGTTGGTCAATTAT
qTUB2-R	CTTAACGATCCAAGTTTATGGATTG
